# Maternal overnutrition programs epigenetic changes in the regulatory regions of hypothalamic *Pomc* in the offspring of rats

**DOI:** 10.1038/s41366-018-0094-1

**Published:** 2018-05-17

**Authors:** Thanuja Gali Ramamoorthy, Tiffany-Jayne Allen, Alison Davies, Erika Harno, Charlotte Sefton, Christopher Murgatroyd, Anne White

**Affiliations:** 1 Faculty of Biology, Medicine and Health, Division of Diabetes, Endocrinology and Gastroenterology, Manchester Academic Health Sciences Centre, University of Manchester, Manchester, M13 9PT UK; 2 School of Healthcare Science, Manchester Metropolitan University, Manchester, UK

## Abstract

**Background and objective:**

Maternal overnutrition has been implicated in affecting the offspring by programming metabolic disorders such as obesity and diabetes, by mechanisms that are not clearly understood. This study aimed to determine the long-term impact of maternal high-fat (HF) diet feeding on epigenetic changes in the offspring’s hypothalamic *Pomc* gene, coding a key factor in the control of energy balance. Further, it aimed to study the additional effects of postnatal overnutrition on epigenetic programming by maternal nutrition.

**Methods:**

Eight-week-old female Sprague–Dawley rats were fed HF diet or low-fat (LF) diet for 6 weeks before mating, and throughout gestation and lactation. At postnatal day 21, samples were collected from a third offspring and the remainder were weaned onto LF diet for 5 weeks, after which they were either fed LF or HF diet for 12 weeks, resulting in four groups of offspring differing by their maternal and postweaning diet.

**Results:**

With maternal HF diet, offspring at weaning had rapid early weight gain, increased adiposity, and hyperleptinemia. The programmed adult offspring, subsequently fed LF diet, retained the increased body weight. Maternal HF diet combined with offspring HF diet caused more pronounced hyperphagia, fat mass, and insulin resistance. The ARC *Pomc* gene from programmed offspring at weaning showed hypermethylation in the enhancer (nPE1 and nPE2) regions and in the promoter sequence mediating leptin effects. Interestingly, hypermethylation at the *Pomc* promoter but not at the enhancer region persisted long term into adulthood in the programmed offspring. However, there were no additive effects on methylation levels in the regulatory regions of *Pomc* in programmed offspring fed a HF diet.

**Conclusion:**

Maternal overnutrition programs long-term epigenetic alterations in the offspring’s hypothalamic *Pomc* promoter. This predisposes the offspring to metabolic disorders later in life.

## Introduction

The prevalence of obesity and other associated metabolic disorders has markedly increased globally, resulting from a complex interaction between multiple factors including genetic, physiological, behavioral and environmental influences. Data from epidemiological and animal studies have suggested that susceptibility to metabolic disorders such as obesity, diabetes, and cardiovascular disease in adulthood can be influenced by early life environment [1]. The fetal period is a critical time for metabolic programming, and therefore the maternal nutritional status during gestation and lactation can determine the onset of metabolic syndrome in offspring [2, 3].

Importantly, fetal programming by maternal diet involves perturbations in hypothalamic neuronal circuits, which are set early in life [4]. The hypothalamic neuropeptides produced by neurons located in the Arcuate nucleus (ARC) have a key role in energy balance regulation [5, 6]. One of the major anorexigenic neuropeptides, α-melanocyte-stimulating hormone, a posttranslational cleavage product of pro-opiomelanocortin (POMC), mediates satiety and controls energy homeostasis [7]. The orexigenic neurons in the ARC primarily release neuropeptide Y (NPY) and agouti-related peptide (AgRP), and have an essential role in increasing food intake [8, 9]. Energy homeostasis is regulated by neurohormonal signals, which are directly related to nutritional state, especially leptin, a key hormone acting on POMC and NPY neurons [10, 11]. Leptin controls *Pomc* gene expression by binding to the long form of the leptin receptor (OB-Rb), leading to activation of signal transducer and activator of transcription 3 (STAT3) by phosphorylation and nuclear translocation. Upon activation, STAT3 binds to the Specificity Protein 1 (SP1)–*Pomc* promoter complex driving *Pomc* transcription to suppress food intake [12, 13]. However, in obesity, leptin resistance is thought to impair leptin signaling [14, 15].

Previous studies from animal models have shown that maternal high-fat (HF) diet and/or obesity increase the susceptibility of offspring to metabolic syndrome [16–23]. Investigations of mechanisms underlying the effect of maternal diet on the offspring’s metabolic homeostasis are limited. Epigenetic modifications especially changes in DNA methylation are thought to mediate the effect by which fetal environment influences adult phenotype and the impact can be transmitted across multiple generations [24–27]. Epigenetic malprogramming of hypothalamic *Pomc* has been implicated in the metabolic programming of obesity by maternal nutrition in offspring [28–31]). However, it is less clear whether these epigenetic modifications are persistent long term, and are affected by the postweaning HF diet. This is an important question as the individuals confronted with altered nutritional status during development are often exposed to a calorie-rich diet later in life.

In this study, we aimed to examine the long-term effect of maternal HF diet exposure on the male offspring’s risk of obesity and to explore whether this was associated with epigenetic changes in the promoter and neuronal enhancer regions of *Pomc*. In addition, we tested for any interactive effects of different combinations of maternal HF and low-fat (LF) diets with postweaning diets that might reflect any (mal) programmed changes in hypothalamic metabolic circuitry following early life overnutrition.

## Materials and methods

### Experimental design

All experiments were performed in accordance with United Kingdom Animals (Scientific Procedures) Act, 1986, using protocols approved by The University of Manchester Ethical Review Panel.

**Fig. 1:**
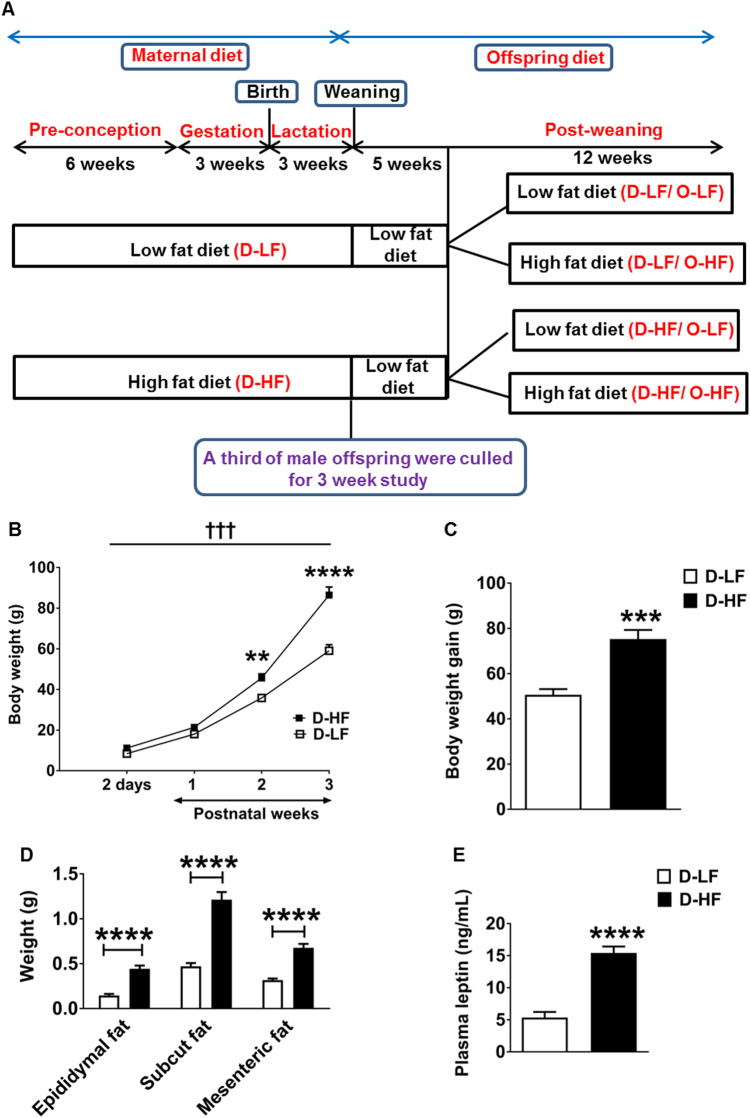
Obesity in male offspring at weaning: **a** experimental outline: female Sprague–Dawley rats (Dams) were fed low- (D-LF) or high-fat (D-HF) diet 6 weeks before conception, and throughout gestation and lactation. On postnatal day (PND) 2, the litter size was reduced to *n* = 10 per dam. On PND 21, one-third of the offspring were culled and the remaining pups from D-HF and D-LF were weaned onto low-fat diet for 5 weeks. At 8 weeks of age, the offspring were divided into groups receiving LF or HF diet until 20 weeks of age. This resulted in four groups of offspring: (1) Dams on LF diet and offspring on LF diet (D-LF/O-LF), (2) Dams on LF diet and offspring on HF diet (D-LF/O-HF), (3) Dams on HF diet and offspring on LF diet (D-HF/O-LF), (4) Dams on HF diet and offspring on HF diet (D-HF/O-HF). **b** Body weight of male offspring of D-LF or D-HF measured from 2 days to 3 weeks of age (two-way ANOVA with repeated measures: maternal diet effect, †††*p* < 0.0002; time effect, *p* < 0.0001; interaction, *p* < 0.0001: D-LF, *n* = 6; D-HF, *n* = 7. Post-hoc Bonferroni **p = 0.0087, ****p < 0.0001). **c** Total Body weight gain of offspring between birth and 3 weeks of age (Student’s *t*-test, D-LF, *n* = 6; D-HF, *n* = 7, ***p = 0.0004). **d** Adipose tissue weights of 3-week-old offspring (Student’s *t*-test, D-LF, *n* = 22; D-HF, *n* = 18) and **e** plasma leptin levels in the offspring recorded at 3 weeks of age (Mann–Whitney *U*-test, *n* = 9, ****p < 0.0001, *Z* = − 3.576, η2 = 0.75). Results are expressed as mean ± SEM.

Eight-week-old female Sprague–Dawley rats (Charles River) were singly housed under a constant 12 h light and dark cycle at an ambient temperature of 23 °C with free access to food and water. A total of 20 female rats were randomly assigned to HF (D-HF) diet (60% of energy from fat; Research Diets D12492) or sucrose-matched control LF (D-LF) diet (10% of energy from fat; Research Diets D12450J). After 6 weeks, they were mated with males, and at day 2 post delivery the litter size was adjusted to 10 pups per dam. Litters from two dams of both the groups were lost (missing postnatally, presumed cannibalized). Body weight and food intake measurements were recorded once a week. At day 21, a third of male pups from LF- and HF-diet-fed dams were culled for the 3 -week study by raising CO_2_ levels followed by cervical dislocation. Fat pads, liver, and skeletal muscle beds were dissected and weighed, and brain was collected and frozen. Blood samples were collected by cardiac puncture and placed in heparinised tubes; plasma was stored at − 80 °C until further analysis. The remaining male pups were weaned onto LF diet for 5 weeks. At 8 weeks of age, the offspring from each group of dams were randomly fed either LF or HF diet resulting in a total of four groups of adult offspring [22] (Fig. 1a). At 8 and 19 weeks of age, an intraperitoneal glucose tolerance test (IPGTT) was performed on each group. Food intake and body weight of offspring were followed until the end of the study. At 20 weeks of age, all groups of offspring were culled, and brain tissue and blood samples were collected as described above.

### Microdissection

Coronal sections of 200 µm thickness were cut from frozen rat brains in a cryostat at approximately − 20 °C, transferred to a microscopic slide, and were frozen immediately. Specific sections from which punches were taken based on the rat brain atlas coordinates of Paxinos and Watson. Punches of specific hypothalamic regions were taken using sample corer, 1 mm diameter (Fine Science Tools), and were pooled for each individual rat. Paraventricular nuclei (PVN) punches were taken from sections between − 1.2 and − 2.4 mm relative to the bregma. ARC punches were taken from adjacent sections between − 2.2 and − 4.2 mm relative to the bregma. Punches were collected in RLT plus buffer containing β-mercaptoethanol from the Qiagen AllPrep DNA/RNA mini kit (80204).

### Analysis of blood parameters

Enzyme-linked immunosorbent assay kits were used to quantify insulin (Crystal Chem 90060) and leptin (Millipore EZRL-83K) in plasma samples. The intra-assay variation for insulin was ≤ 10% and for leptin it was 2.4% Blood glucose levels were measured by Accu-Chek Glucometer (Roche). The homeostatic model assessment for insulin resistance (HOMA-IR) was calculated as follows: fasting serum insulin concentration (µU/ml) multiplied by fasting blood glucose levels (mg/dl) divided by 405 [32].

### Intraperitoneal glucose tolerance test

IPGTT was performed on offspring at 8 and 19 weeks of age. Animals were kept under fasting conditions for 16 h and injected intraperitoneally with 2 g of glucose per kg body weight. Tail vein blood was collected at baseline and at 15, 30, 60, and 90 min after glucose administration for 8-week-old offspring and at baseline, 15, 60, and 90 min after glucose administration for 19-week-old offspring. Accu-Chek Glucometer was used to measure blood glucose concentration.

### Quantitative reverse-transcription PCR

Total RNA was isolated using Qiagen RNeasy micro kit (74004) according to the manufacturer’s instructions. Reverse transcription was conducted with 250 ng of total RNA using GoScript^TM^ Reverse Transcription system (Promega A5001). The quantitative PCR (qRT-PCR) was performed using cDNA, KAPA SYBR^®^ FAST qPCR kit Master Mix 2 × (KK4602) universal, and forward and reverse primers (sequences shown in Supplementary Table 1) in the Applied Biosystems real-time PCR instrument. *Hprt* was employed as a housekeeping gene. Relative mRNA expression was calculated using the standard curve method. We analyzed the mRNA levels of energy homeostasis markers (*Npy*; *AgRP*; Pomc and leptin receptor, Ob-Rb in the arcuate nuclei of the hypothalamus; melanocortin receptor, *Mc4r* and neuropeptide y receptor Y1, *Npy1r* in the paraventricular nuclei of hypothalamus), the expression of DNA methyltransferases 1 *Dnmt1* and *2a*, the mRNA levels DNA methyl group-binding proteins (methyl CpG-binding protein 2 *Mecp2*; methyl CpG-binding domain protein 2, *Mbd2*, and *Sp1*) in the arcuate nuclei of the hypothalamus. The expression of all target genes, when normalized to another internal control, β-actin (*Actb*), was similar.

### DNA methylation analysis

Genomic DNA from ARC punches were isolated using Qiagen AllPrep DNA/RNA mini kit (80204) according to the manufacturer’s instructions. The bisulfite conversion was carried out using EpiTect Fast DNA bisulfite kit (59824). *Pomc* promoter region was amplified with the forward primer 5′-GTTTAGTTTTGAGTGGAGATTTAATAGTA-3′ and the reverse primer 5′-TCCCTATCACTCTTCTCTCTTCTTTTA-3′. *Pomc* npe1 enhancer region was amplified with three sets of primers forward primer1 5′-GTTTTAGTTGGGGTTTAGTGTTATTTA-3′ and the reverse primer 5′-TATCCCAACTACCTAAAATCCTCTAC-3′, forward primer2 5′-TTTTATTGTGGGGTTAGTAGTAGG-3′ and the reverse primer2 5′- CAAAAATACAAAATTCTCCAACAAAATCTA-3′, forward primer3 5′-GGGTTTATTGTGGTTTTTATTGAGTTAGAT-3′ and the reverse primer3 5′-TATACCCTTTCCCAAACTTACCTTA-3′. *Pomc* npe2 enhancer region was amplified with forward primer 5′-TGGTGGGTTGTTGTGTTAATAT-3′ and the reverse primer 5′-AACCCCTTTATAACATTCAATATAACCTC-3′. The reverse primers are biotinylated. PCR amplification of the genomic fragment of *Pomc* promoter of 290 bp and enhancer regions was performed using Qiagen Pyromark PCR kit (catalogue number 978703) at the following conditions: initial PCR activation step at 95 °C for 15 min followed by 40 cycles of (denaturation at 94 °C for 30 s, annealing at 56 °C for 30 s, and extension at 72 °C for 30 s) and final extension at 72 °C for 10 min. The PCR products were then captured using streptavidin-coated agarose beads (Streptavidin Sepharose High Performance beads, GE Healthcare) under denaturing conditions to obtain single-stranded DNA. The pyrosequencing reaction was then carried out using the Pyromark Q24 system (Qiagen) and Pyromark Q24 Advanced CpG Reagents (970922). The sequencing primers used for *Pomc* promoter region were S1: 5′-GTATTTTTAATTAAGTTTTTTTTGA-3′, S2: 5′-ATTTTTTAGGTATATTTGTTG-3′, and S3: 5′-GGAAGTTTTTTTT-3′; for nPE1 region were nPE1 S1: 5′-GGGGTTTAGTGTTATTTATT-3′, nPE1 S2: 5′-GTAAGTTTGAGTTTTGAATG-3′, nPE1 S3: 5′-ATTGAGTTAGATTGGTGA-3′; and for nPE2 was 5′-GGTTTTTTGGTTGTAATAAAGT-3′.

### Statistical analysis

No blinding was carried out for data analysis. Data were analyzed with GraphPad Prism 7.0 and IBM SPSS Statistics 20. The number of animals per group was established according to the common practice in molecular biology (*n* = 5–8) and phenotypic analysis (*n* = 12–16). The exact *n* used for each group is indicated in the figure legends. Normality was assessed using the Shapiro–Wilks test. For multiple group comparisons, Levene’s test was used to determine the equality of variances between the groups. If the variance was heterogeneous, data were log transformed before analysis. Statistical outliers were detected using Grubb’s test (*P* < 0.05). Comparisons between two groups were performed by Student’s *t*-test or Mann–Whitney *U*-test if the data are not normally distributed. Body weight in 3-week-old offspring was analyzed by two-way analysis of variance (ANOVA) with repeated measures followed by Bonferroni post-hoc analysis. Two-way ANOVA followed by Tukey *post-hoc* analysis was used to compare the mean differences between multiple groups. Body weight and calorific intake in 20-week-old offspring were analyzed by three-way ANOVA with repeated measures followed by Bonferroni post-hoc analysis. The effect size between two groups for leptin data are given as η2 and for insulin data between four groups are given as ηp2. *P* < 0.05 was considered significant. Data are presented as mean ± SEM.

## Results

### Maternal HF diet feeding predisposes the offspring to metabolic disorders at weaning

To determine the effects of maternal HF diet feeding on epigenetic programming of obesity in the offspring, we fed 8-week-old female Sprague–Dawley rats either a HF diet or a control LF diet for 6 weeks before conception, then throughout gestation, and finally during lactation (Fig. 1a). Rats on HF diet became significantly heavier after 6 weeks than the LF-diet-fed control animals (Supplementary Figure 1A). They were mated after 6 weeks on the obesogenic diet at which point they had ~ 10% increased body weight compared with the LF-diet-fed rats. The difference in body weight was maintained in the first 2 weeks of pregnancy (Supplementary Figure 1A). Calorific intake from fat was significantly increased throughout the period of diet challenge (Supplementary Figure 1B). During lactation, the body weight of dams from both groups was similar, albeit with a significant increase in the average daily total caloric intake in the HF-diet-fed group (Supplementary Figure 1, A and C). Moreover, the mesenteric and subcutaneous fat content and plasma leptin levels were similar between the groups of dams at the end of lactation (Supplementary Figure 1, D and E).

Exposure to maternal overnutrition resulted in body weight increasing by 32% in the male pups (D-HF) compared with the control pups (D-LF) at 2 days after birth (Fig. 1b). At 3 weeks of age, maternal HF diet consumption resulted in a significant increase in body weight [49%] compared with control offspring (Fig. 1c). This was attributed to an increase in both fat mass [epididymal, mesenteric, as well as subcutaneous fat weight (Fig. 1d)] and non-fat mass [liver D-LF, 2.34 ± 0.08 vs. D-HF, 4.11 ± 0.16, *P* < 0.0001; gastrocnemius muscle D-LF, 0.21 ± 0.01 vs. 0.38 ± 0.02, *P* < 0.0001; quadriceps muscle D-LF, 0.25 ± 0.01 vs. D-HF, 0.36 ± 0.01, *P* < 0.0001; D-LF, *n* = 22 and D-HF, *n* = 20]. Obesity in D-HF pups was associated with hyperleptinemia (Fig. 1e). Thus, the characteristic features of metabolic syndrome had already occurred at 3 weeks of age.

### Effects of maternal HF diet feeding on the expression of hypothalamic markers of energy balance in the offspring at 3 weeks of age

**Fig. 2:**
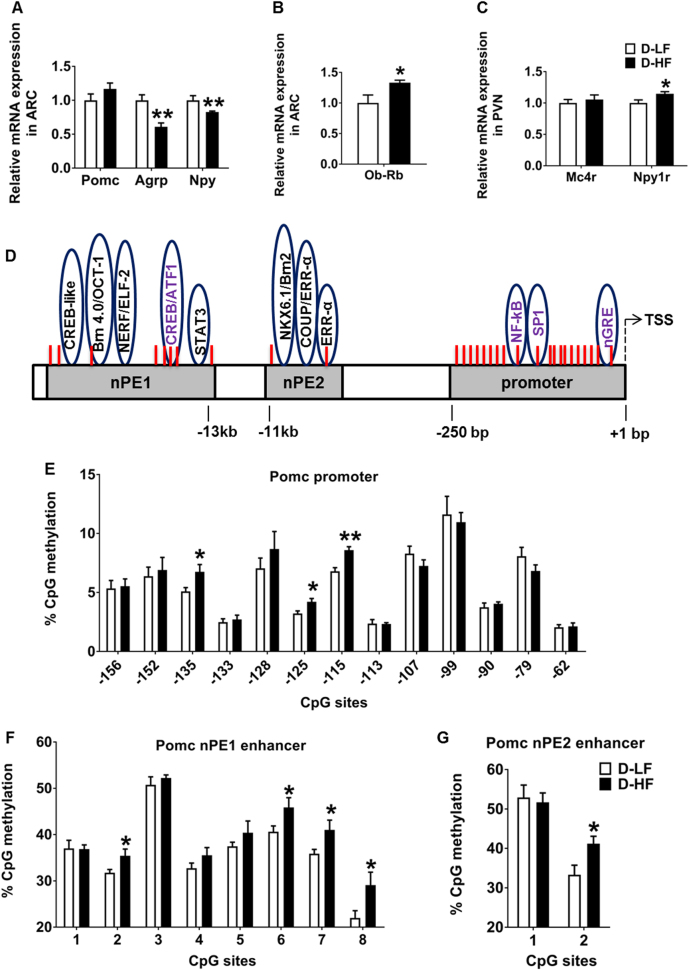
Gene expression and hypothalamic *Pomc* DNA methylation changes in offspring at weaning. **a** mRNA expression levels of *Pomc*, *Agrp*, *Npy*, and **b** *Ob-Rb* in the ARC. **c** Relative mRNA levels of *Mc4r* and *Npy1r* in the PVN analyzed by qRT-PCR in the 3-week-old offspring of LF- or HF-fed dams (Student’s *t*-test, *n* = 8). **d** Map of the Pro-opiomelanocortin (*Pomc*) gene promoter and enhancer region including functional regulatory elements and CpG dinucleotides (red lines). **e** Methylation analyzes of hypothalamic *Pomc* promoter (− 150 bp to transcription start site [TSS]) (Student’s *t*-test, D-LF, *n* = 6; D-HF, *n* = 7) and **f**, **g** of neuronal *Pomc* enhancer region 1 and 2 in the offspring of LF- or HF-fed mothers at 3 weeks of age (Student’s *t*-test, *n* = 8). Data are shown as mean ± SEM. *p < 0.05, **p < 0.01. *Pomc*, pro-opiomelanocortin; *Agrp*, agouti-related peptide; *Npy*, neuropeptide Y; *Ob-Rb*, long form of the leptin receptor; *Mc4r*, melanocortin 4 receptor; *Npy1r*, neuropeptide Y receptor Y1

To understand the mechanisms underlying the obese phenotype in the programmed offspring, we first determined the expression of neuropeptides critically involved in the control of energy and glucose homeostasis. The expression of orexigenic *Agrp* and *Npy* was significantly decreased in the ARC punches of D-HF offspring at 3 weeks of age (Fig. 2a). This could be an adaptation to overcome the effects of over-nourishment early in life. No difference in the expression of the orexigenic genes, *Galanin*, *Enkephalin*, and *Dynorphin* was detected in the PVN punches of offspring (Supplementary Figure 2A). Despite the increase in body weight and adiposity, an increase in the expression of anorectic *Pomc* mRNA was not observed in the D-HF offspring (Fig. 2a). When both the groups were combined, plasma leptin levels negatively correlated with *Npy* and *AgRP* mRNA expression, whereas no correlation with *Pomc* expression was observed (*r* = − 0.5670 for *Npy*, *P* = 0.02; *r* = − 0.5974 for *Agrp*, *P* = 0.01; *r* = 0.2331 for *Pomc*, *P* = 0.4; *n* = 16). The increase in plasma leptin levels was accompanied by augmented expression of the long form of leptin receptor (*Ob-Rb*) in the ARC of D-HF offspring (Fig. 2b). In the PVN, expression of the Y1 receptor, the major NPY receptor in the PVN (*Npy1r*) was upregulated slightly by maternal HFD consumption, which could lead to altered energy expenditure and predisposition to increased food intake. Expression of PVN *Mc4r*, known to be involved in feeding regulation, did not differ in expression between the groups (Fig. 2c).

### Maternal overnutrition programs hypermethylation of the hypothalamic *Pomc* promoter and enhancer regions in the offspring at 3 weeks of age

Circulating leptin is known to stimulate the expression of the anorexigenic *Pomc* gene, while inhibiting the orexigenic *Npy* and *Agrp* [33–35]. The increase in plasma leptin levels observed in the D-HF offspring did not induce the expression of ARC *Pomc*. We hypothesized that this could be due to the altered methylation status in the regulatory regions controlling *Pomc* expression. To this end, we analyzed the methylation status of 20 CpGs spanning − 238 to − 62 bp of the *Pomc* promoter, which includes DNA-binding sequences of transcriptional regulatory elements essential for the regulation of *Pomc* gene expression [36] (Fig. 2d). No difference in methylation at CpG sites from − 238 to − 164 bp was observed between the groups (Supplementary Figure 4A). However, an increase in methylation at CpG sites near the SP1-binding site (− 135 [30%], − 125 [31%], and − 115 [26%]) was observed in the *Pomc* promoter of D-HF offspring at 3 weeks of age (Fig. 2e). The Sp1-binding site is essential for the leptin-mediated activation of *Pomc* by the Sp1-STAT3 binding to the promoter [13] and methylation of this site abrogates the binding of Sp1 [37, 38]. Given that Sp1 occupying its binding site could prevent methylation of the site [39], we checked the mRNA expression of *Sp1* as decreased levels might explain the increased methylation. However, there was no change in *Sp1* mRNA levels at 3 weeks (Supplementary Figure 3B). Therefore, increased methylation at this site is not as a result of shortage of Sp1. The expression levels of methyltransferases *Dnmt1* and *Dnmt3b*, and methyl CpG-binding proteins *Mecp2* and *Mbd2* also did not differ between the groups in 3-week-old offspring (Supplementary Figure 3A).

*Pomc* transcription in the hypothalamus is governed by the two enhancer regions nPE1 and nPE2 located ~ 10 to 12 kb upstream of the transcriptional start site (TSS) [40]. To determine whether maternal nutrition affects the methylation status of these enhancer regions, we mapped the percentage methylation levels of CpG sites in the hypothalamic *Pomc* nPE1 and 2 regions. The nPE1 enhancer spans 600 bp including eight CpG dinucleotides. The sequence covers binding sites for transcription factors Stat3, Brn 4.0/OCT-1, NERF/ELF2, and CREB/ATF1 [40] (Fig. 2d). The nPE2 enhancer spans 150 bp and includes two CpG sites covering binding sites for NKX6.1/Brn2, COUP/ERRα, and ERRα [40] (Fig. 2d). Analysis showed that nPE1 had enhanced methylation at CpG sites 2, 6, 7, and 8 in ARC punches of D-HF offspring compared with D-LF control animals. Moreover, CpG site 2 in the nPE2 region is also hypermethylated in D-HF offspring compared with the controls (Fig. 2f,g). Taken together, these results show that the DNA methylation status of *Pomc* regulatory regions can undergo dynamic changes in the offspring in response to maternal HF diet feeding, and that a higher level of DNA methylation may therefore impair *Pomc* expression in hyperleptinaemic D-HF offspring.

### Metabolic disorders at 3 weeks of age persisted through adulthood

**Fig. 3:**
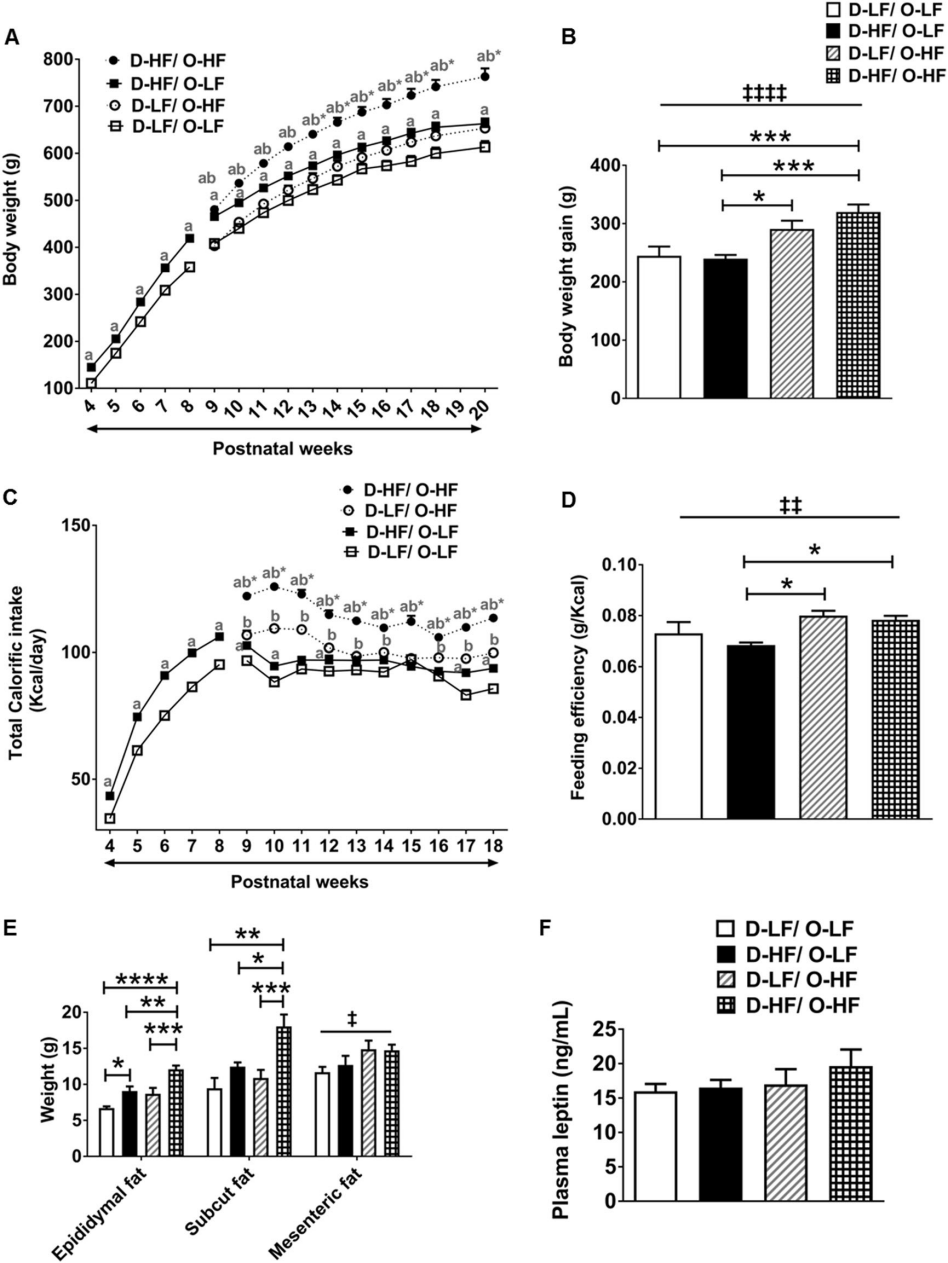
Obesity in adult male offspring. **a** Body weight of offspring from LF- and HF-fed dams who were weaned onto LF diet for 5 weeks and then fed either LF or HF diet from week 8 until week 20 (three-way ANOVA with repeated measures: maternal diet effect, *p* < 0.0001; postweaning diet effect, *p* < 0.0001; time effect, *p* < 0.0001; time × maternal diet, *p* = 0.0001; postweaning diet × maternal diet interaction, *p* < 0.0001; *n* = 12 per group except D-HF/O-HF group, *n* = 16. Post-hoc Bonferroni, ab*, *p* < 0.0001 compared with all other groups; ab, *p* < 0.0001 compared with D-LF/O-LF and D-LF/O-HF; **a**, *p* < 0.01 compared with D-LF/O-LF). **b** Body weight gain from 8 to 20 weeks of age (two-way ANOVA: postweaning diet effect, ^‡‡‡‡^*P* < 0.0001; post-hoc Tukey’s test, *n* = 12 per group except D-HF/O-HF group, *n* = 16). **c** Average daily calorific intake of offspring (three-way ANOVA with repeated measures: maternal diet effect, *p* < 0.0001; postweaning diet effect, *p* < 0.0001; time effect, *p* < 0.0001; time × maternal diet interaction, *p* = 0.0161; time × postweaning diet interaction, *p* < 0.0001; maternal diet × postweaning diet interaction, *p* < 0.0001; time × maternal diet × postweaning diet interaction, *p* = 0.0181; *n* = 12 per group except D-HF/O-HF group, *n* = 16. Post-hoc Bonferroni, ab*, *p* < 0.0001 compared with all other groups; **a**, *p* < 0.05 compared with D-LF/O-LF; **b** *p* < 0.001 compared with D-LF/O-LF). **d** Feeding efficiency of offspring fed with either LF or HF diet for 12 weeks (two-way ANOVA: postweaning diet effect, ^‡‡^*P* = 0.0013; post-hoc Tukey’s test, *n* = 12 per group except D-HF/O-HF group, *n* = 16). **e** Epididymal fat pad weight (two-way ANOVA: maternal diet effect, *P* < 0.0001; postweaning diet effect, *P* < 0.0001; post-hoc Tukey’s, *n* = 12 per group except D-HF/O-HF group, *n* = 16), Subcutaneous fat pad weight (two-way ANOVA: maternal diet effect, *P* = 0.0002; postweaning diet effect, *P* = 0.0258; post-hoc Tukey’s, D-LF/O-LF, *n* = 5; D-HF/O-LF, *n* = 12; D-LF/O-HF, *n* = 12; D-HF/O-HF, *n* = 16) and mesenteric fat pad weight (two-way ANOVA: postweaning diet effect, ^‡^*P* = 0.0107: D-LF/O-LF, *n* = 12; D-HF/O-LF, *n* = 12; D-LF/O-HF, *n* = 10; D-HF/O-HF, *n* = 16). **f** Plasma leptin levels in the offspring at week 20 (*n* = 10 per group except D-HF/O-HF group, *n* = 8). Data are shown as mean ± SEM. *P < 0.05, **P < 0.01, ***P < 0.001, ****P < 0.0001

We aimed to determine whether the programming of metabolic disorders observed at 3 weeks persists through adulthood and also to study the additional effects of postweaning diet in the offspring. To this end, each group of offspring was exposed to either a LF diet (D-LF/O-LF and D-HF/O-LF) or HF diet (D-LF/O-HF and D-HF/O-HF) from 8 weeks until the age of 20 weeks (Fig. 1). Post weaning, the male offspring from dams on HF diet, which were given a LF diet (D-HF/O-LF) had a higher body weight than the D-LF/O-LF group but then just maintained this increased body weight (Fig. 3a) so that the body weight gain from week 8 to week 20 was similar for both groups (Fig. 3b). The food intake of D-HF/O-LF was significantly higher than the control offspring on the same diet (Fig. 3c). The higher calorific intake was required to maintain the body weight increase as shown by similar feed efficiency [ratio of total body weight gained (g) to energy consumed (kJ)] compared with D-LF/O-LF group (Fig. 3d). These results indicate that the programming of obesity by maternal HF diet that occurred at 3 weeks persists until 20 weeks of age and that D-HF offspring weaned to LF diet failed to return to lean energy balance throughout their life.

Upon HF diet challenge, adult offspring of HF-diet-fed dams gained significantly more weight and consumed more calories compared with offspring of control dams (Fig. 3a–d). Moreover, the mass of epididymal and subcutaneous fat pads was significantly more in the D-HF/O-HF offspring compared with the other groups (Fig. 3e). Therefore, HF feeding exacerbated the programming effects observed in the maternally overfed offspring. In addition, postweaning HF diet led to an increase in mesenteric fat pad weight in both the groups of offspring with differing maternal diet (Fig. 3e). However, plasma leptin levels were similar across the different groups (Fig. 3f).

**Fig. 4:**
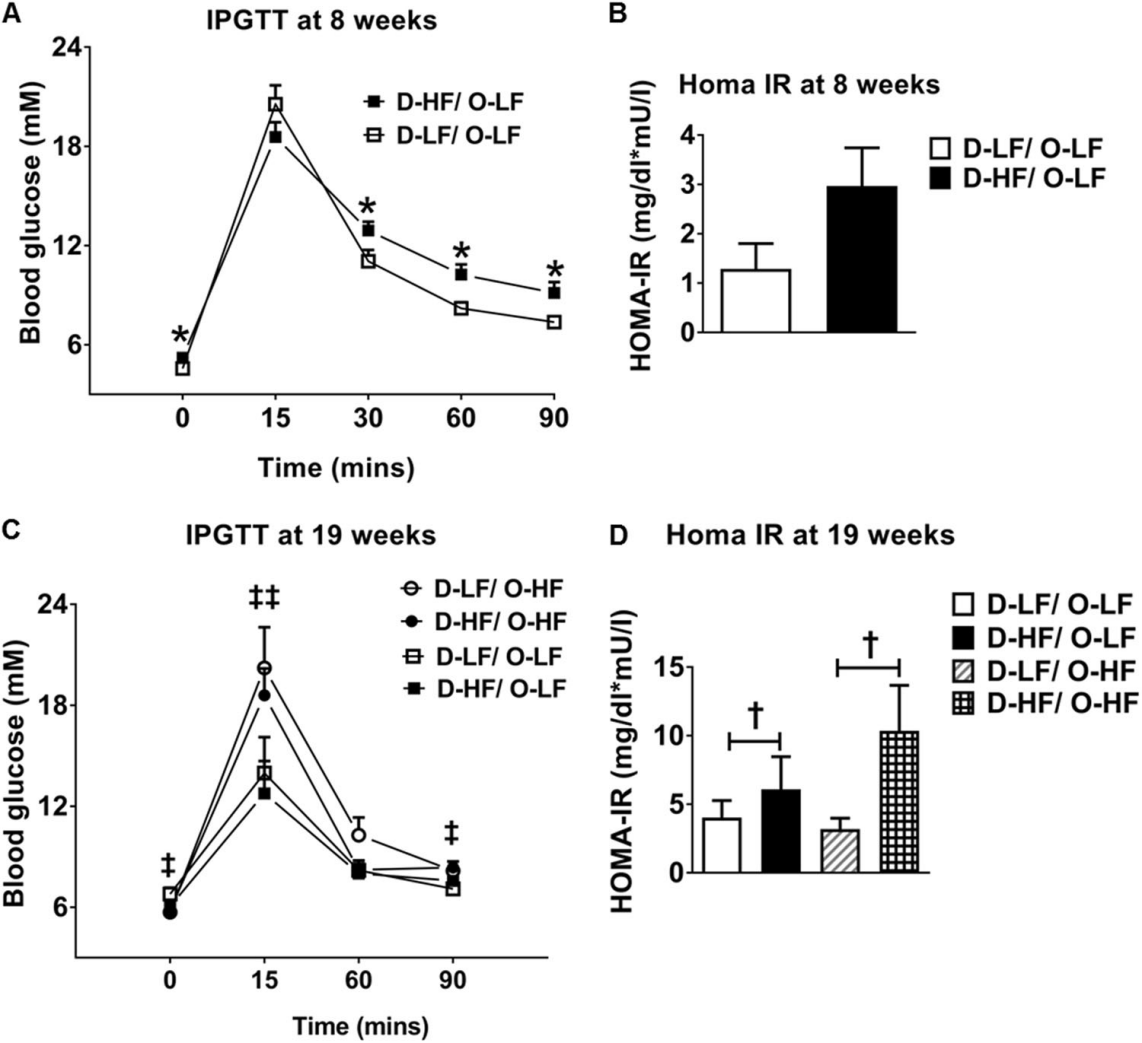
Glucose homeostasis in the adult male offspring. **a** Intraperitoneal glucose tolerance test (IPGTT) at 8 weeks (Student’s *t*-test, *n* = 7) and **c** at 19 weeks of age (two-way ANOVA: postweaning diet effect at t0, ^‡^*P* = 0.0436; t15, ^‡‡^*P* = 0.0077; t90, ^‡^*P* = 0.0168, *n* = 8) in the offspring of LF- or HF-fed dams. **b**,**d** homeostatic model assessment indices of insulin resistance (HOMA-IR) in the offspring of LF- or HF-fed dams at week 8 (D-LF, *n* = 5; D-HF, *n* = 6) and at week 19 (two-way ANOVA: maternal diet effect, ^†^*P* = 0.0495, *n* = 5, ηp^2^ = 0.22). Data are shown as mean ± SEM. *P < 0.05

Maternal HF diet led to higher fasting blood glucose levels and glucose intolerance in the offspring at 8 weeks of age (Fig. 4a) associated with a tendency toward increased HOMA-IR (Fig. 4b). However, these effects on glucose homeostasis were corrected at 19 weeks of age (Fig. 4c). Although postweaning HF diet caused increased glucose intolerance in both the groups of offspring with differing maternal diet, insulin resistance was only observed in the maternally overfed offspring compared with the offspring of LF-fed dams (Fig. 4c,d). Thus, exposure of mothers to HF diet programs alterations in energy and glucose homeostasis in male offspring with postweaning HF diet exacerbating some of the effects.

### Expression of hypothalamic markers of energy balance regulation in the adult offspring

**Fig. 5:**
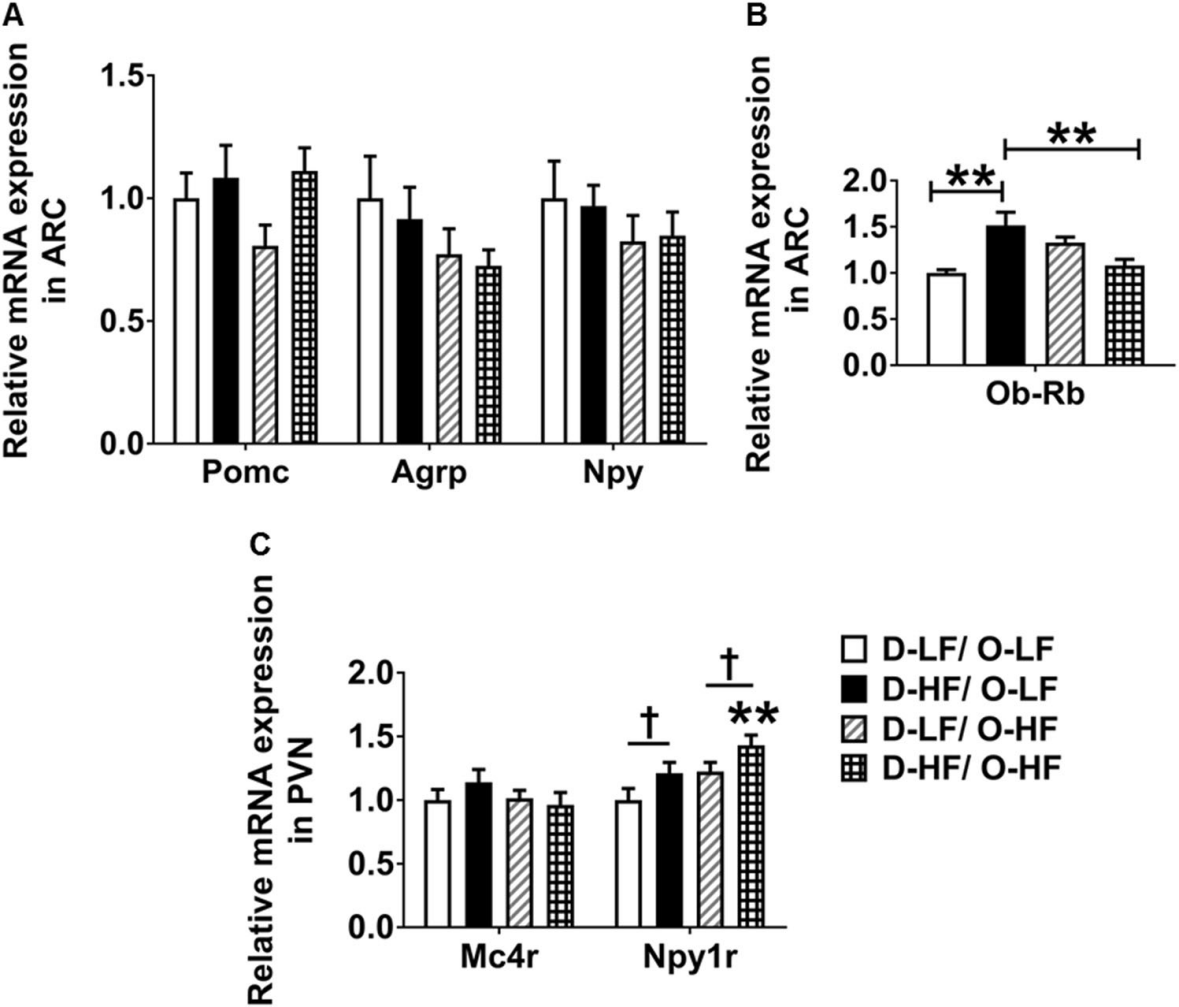
Mean relative gene expression levels in the ARC and PVN punches for 20-week-old offspring. **a** mRNA expression levels of *Pomc*, *Agrp*, *Npy* in the ARC. **b** Relative mRNA levels of *Ob-Rb* in the ARC (two-way ANOVA: maternal diet × postweaning diet interaction, *P* = 0.0002; post-hoc Tukey’s test, *n* = 6). **c** Relative mRNA levels of *Mc4r* in the PVN analyzed by qRT-PCR in the offspring at week 20 and *Npy1r* (two-way ANOVA: †maternal diet effect, *P* = 0.0136; ‡postweaning diet effect, *P* = 0.0192; post-hoc Tukey’s test, **P < 0.01 compared with D-LF/O-LF, *n* = 6). Data are shown as mean ± SEM. *P < 0.05, **P < 0.01

As observed at 3 weeks, the mRNA expression of ARC *Pomc*, PVN *Mc4r*, and PVN orexigenic genes is unaltered by maternal HF diet and by the postweaning HF diet feeding (Fig. 5a,c and Supplementary Figure 2B). The decrease in *Agrp* and *Npy* expression as a result of maternal overnutrition that occurred at 3 weeks did not persist through adulthood (Fig. 5a). Similarly, the postweaning HF diet did not have any effect on the expression of these genes (Fig. 5a,c and Supplementary Figure 2B). However, the PVN Y1 receptor (*Npy1r*) mRNA was induced by maternal HF diet and exacerbated by offspring’s HF diet consumption. This could contribute to the orexigenic effects leading to maintenance of increased body weight in the programmed offspring (Fig. 5c). Although leptin levels were similar between the groups at 20 weeks, the increased expression of the long form of leptin receptor (*Ob-Rb*) in the ARC has been maintained in the D-HF/O-LF adult offspring (Fig. 5b).

### Hypermethylation in the Pomc promoter but not enhancer region persists in the adult offspring

**Fig. 6:**
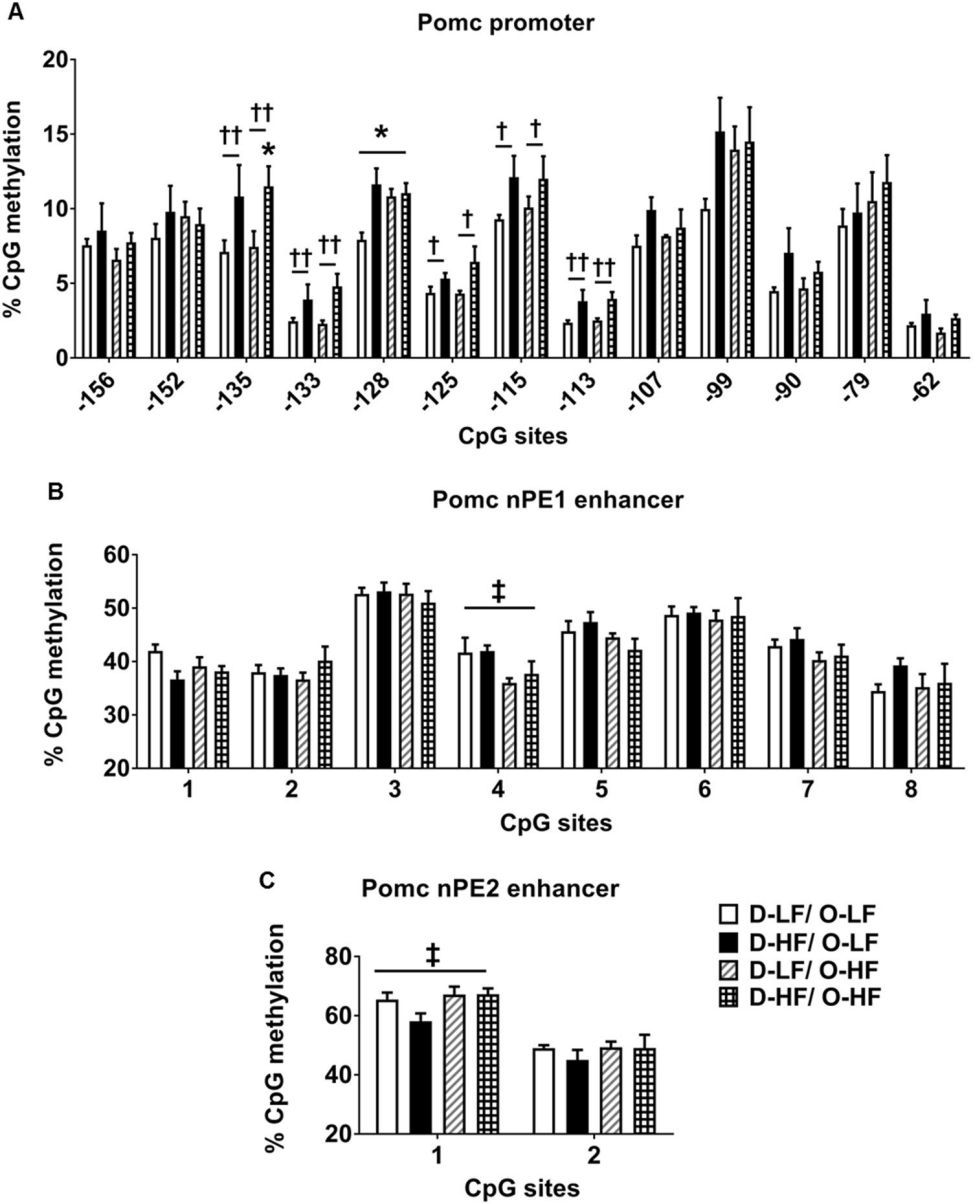
DNA methylation changes at hypothalamic *Pomc* regulatory regions in the adult offspring. **a** Methylation analyzes of hypothalamic *Pomc* promoter (− 150 bp to TSS) − 135 site (two-way ANOVA: maternal diet effect, ^††^*P* = 0.0037; post-hoc Tukey’s test, *P < 0.05 compared with D-LF/O-LF), − 133 site (two-way ANOVA: maternal diet effect, ^††^*P* = 0.0086), − 128 site (two-way ANOVA: maternal diet effect, ^†^*P* = 0.0122; maternal × postweaning diet interaction, *P* = 0.0228; post-hoc Tukey’s test, *P < 0.05 compared with D-LF/O-LF), − 125 site (two-way ANOVA: maternal diet effect, ^†^*P* = 0.0280), − 115 site (two-way ANOVA: maternal diet effect, ^†^*P* = 0.0473), − 113 site (two-way ANOVA: maternal diet effect, ^††^*P* = 0.0042) (*n* = 6 for D-LF/O-LF and D-HF/O-HF, *n* = 5 for D-HF/O-LF and D-LF/O-HF). **b**,**c** Methylation analyzes of neuronal POMC enhancer region 1 and 2 in the adult offspring fed postnatally LF or HF diet nPE1 CpG 4 (two-way ANOVA: postweaning diet effect, ^‡^*P* = 0.0185; *n* = 6), nPE2 CpG 1 (two-way ANOVA: postweaning diet effect, ^‡^*P* = 0.0469; *n* = 5). Data are shown as mean ± SEM. *P < 0.05, **P < 0.01

To evaluate whether hypermethylation of the *Pomc* promoter and enhancer is conserved in the offspring of HF-fed dams from weaning to adulthood and to study the effects of postweaning HF diet, we analyzed the methylation pattern in 20-week-old offspring fed LF or HF diet. As observed at 3 weeks, the hypermethylation near SP1 binding sites − 135 [52%], − 125 [22%], and − 115 [30%] was maintained through adulthood. Interestingly, increase in the methylation at additional sites − 202 [19%], − 133 [59%], − 128 [47%], − 125 [22%], and − 113 [61%] was observed in the adult offspring as a result of maternal overnutrition (Fig. 6a). These results indicate that this methylation pattern established at critical time periods is stable and is associated with long-term effects on regulation of metabolic homeostasis. Post-weaning overnutrition induced hypermethylation in the control offspring at site − 128 [37%] but did not have any additive effects on the methylation levels near Sp1-binding sites in the programmed offspring (Fig. 6a). However, HF feeding caused hypermethylation at an additional site in the distal region (− 224) in programmed offspring compared with controls (Supplementary Figure S4). Surprisingly, the percentage methylation levels at CpG sites − 238 and − 224 in the adult offspring were slightly lower compared with those at 3 weeks of age, although at other sites age-dependent increases in percentage methylation were observed (Supplementary Figure S4).

Maternal HF feeding resulted in an increase in percentage methylation at *Pomc* enhancer regions nPE1 and nPE2 in 3-week-old pups (Fig. 2f,g). However, these changes did not continue through adulthood (Fig. 6b,c). In fact, there was a postweaning HF-diet-induced decrease in methylation at CpG site 4 in nPE1 region (Fig. 6b).

## Discussion

This study examined the impact of maternal obesity on central neuroendocrine circuitry and metabolic homeostasis in the offspring at weaning. Moreover, we investigated whether the effects were made worse in adult offspring exposed to a HF diet. We demonstrated that maternal HF diet predisposes these male offspring to early-onset obesity. At weaning, the offspring had higher circulating leptin levels. This was associated with hypermethylation in both the *Pomc* promoter and enhancer regions, which affect leptin-mediated increases in hypothalamic *Pomc* expression. Importantly, the increase in body weight and *Pomc* promoter hypermethylation persisted even at 20 weeks. This highlights the long-term influence of maternal diet on the offspring’s epigenetic phenotype related to impaired energy homeostasis. In addition, programmed offspring were more vulnerable to a postweaning HF diet, which induced increased caloric intake, adiposity, and insulin resistance. However, postweaning HF diet did not have additive effects on changes in methylation levels.

There is clear evidence from this study that maternal obesity before and during pregnancy programs weight gain in the pups in that they were 32% heavier than control pups at 2 days after birth. There was also a 49% increase in body weight gain in programmed offspring from birth to 3 weeks. The increased weight gain in the offspring was associated with hyperleptinemia. Leptin has been shown to stimulate the expression of anorexigenic *Pomc*, while downregulating the orexigenic *Agrp* and *Npy* mRNA expression [33–35]. This increase in leptin would be expected to increase the mRNA expression of *Pomc*, leading to reduced food intake and decreased body weight. Although we observed an adaptive decrease in *Agrp* and *Npy* mRNA levels, programmed offspring presented with no change in the mRNA expression of *Pomc*. These results suggest that *Pomc* expression is not responsive to high levels of leptin in these animals.

We hypothesized that this failure in the homeostatic adaptive change in *Pomc* could be due to increased DNA methylation. Exposure to unbalanced nutrition in utero induces persistent gene-specific DNA methylation changes [41–44]. Indeed, in this study we found hypermethylation at the transcription factor-binding sites for Sp1-Stat3 on the *Pomc* promoter that are associated with leptin signaling. Other developmental programming models have shown hypermethylation near Sp1-binding sites in the *Pomc* promoter [29, 36, 37], suggesting that this could be a common pathway for different programming paradigms. In addition, hypermethylation in the Sp1-binding site in the offspring’s hypothalamic *Pomc* has been shown to decrease Sp1 binding to its cognate sites [37] Therefore, in our model of maternal obesity, the increased methylation in the regulatory region of *Pomc*, which governs its leptin-mediated expression, could be contributing to the mechanism causing resistance to leptin and to the increased body weight. Development of leptin resistance is very complex and varies between models. Although leptin resistance is documented in diet-induced obesity models as well as models of monogenic or polygenic obesity, the underlying mechanisms have not been fully determined [45]. Although resistance can occur at the level of receptor signaling, there is no mechanism that fits all paradigms. In addition, the absolute role of leptin resistance in the gain of body weight or the maintenance of obesity has been queried. Instead, it has been suggested that it is leptin levels that are important and this is in situations where they should be considered more as a starvation signal [46]. Therefore, it is perhaps not surprising that for developmental programming there could be another mechanism such as *Pomc* methylation that imparts a longer term block on *Pomc* transcription.

The perinatal period is considered to be a critical window of development and exposure to adverse conditions during this time can alter the development trajectory of the offspring and impact on its long-term health and behavior [47–49]. For this reason, at the adult stage, we were interested to determine whether the epigenetic programming by maternal diet was maintained or reprogrammed. Clearly, in the current study, the increase in body weight and adiposity programmed by maternal HF diet was maintained through to adulthood in rats up to 20 weeks of age. This exposure to maternal HF diet also led to hyperphagia in the offspring throughout their life. More importantly, the hypermethylation at the *Pomc* promoter was evident in adulthood, demonstrating the persistent nature of this effect in the hypothalamus. This emphasizes the enduring nature of early environmental programming effects. Thus, the differences in the metabolic phenotype observed in the adult offspring associated with the maternal nutritional imbalance during early life could be mediated by the sustained DNA methylation changes.

An interesting finding from our study is that altered perinatal nutritional exposure exacerbates the sensitivity to the obesogenic environment in later life. The programmed offspring were more vulnerable to eating HF diet in that the obese phenotype was exacerbated and induced insulin resistance. These results indicate that maternal HF diet consumption predisposes the offspring to obesity and it may be because they are programmed to have increased susceptibility to a postweaning HF diet. However, the increase in size of adipose tissue depots with postweaning HF diet occurred without any increase in leptin concentrations in the adult offspring. Similar observations have been made in previous studies [50]. This may be because of other regulatory mechanisms in adipose tissue that affect leptin expression. The discordance between increased adiposity and lack of an increase in leptin levels may be further indication that leptin does not act necessarily to decrease body weight gain. Therefore, mechanisms such as methylation of the *Pomc* promoter are more likely to be important.

Post-weaning HF diet consumption did not have any additive effects on the methylation levels near Sp1-binding sites, although it caused hypermethylation at an additional site (− 224) in the programmed adult offspring. These results indicate that DNA methylation levels in *Pomc* regulatory regions are more susceptible to changes in nutritional exposure during development than in mature rats. In other studies not related to developmental programming, HF diet feeding in adult rats is associated with an increase in DNA methylation in the SP1-binding site of *Pomc* promoter and decreases the expression of *Pomc* [38]. However, in our HF-fed adult rats, we did not observe any difference in *Pomc* mRNA. This could be due to differences in the timing and duration of exposure to HF diet.

The enhancer regions (nPE1 and nPE2) in *Pomc* located about 10–12 kb upstream of the TSS are phylogenetically conserved both in terms of nucleotide sequence and organization within the locus in placental animals [40, 57–59]. These two neuronal enhancers are necessary to drive hypothalamic expression [40]. On the other hand, the pituitary expression of *Pomc* is dependent on the proximal promoter and an enhancer located 7 kb upstream of the TSS [60]. To date, there is no information on methylation in these enhancer regions and whether it is subject to developmental plasticity. For the first time, we have shown that methylation of the *Pomc* hypothalamic-specific enhancers (both nPE1 and nPE2) is influenced by early life nutritional exposure in rat pups. The nPE1 enhancer contains a conserved canonical site for Stat3 binding and therefore could have a role in leptin regulation of *Pomc* expression [40]. Interestingly, in the programmed offspring at weaning, we observed hypermethylation in the CpG site close to the Stat3-binding site, as a result of maternal overnutrition. This implies an adaptive plasticity in epigenetic modulations in *Pomc* enhancer regions during development. Surprisingly, we observed that methylation levels at this enhancer region were normalized to that of controls in adulthood and were not influenced by an obesogenic diet in later life. These results suggest that epigenetic modifications in the enhancer region may not be directing the mechanisms behind the phenotypic differences in adults. Therefore, persistent promoter hypermethylation is probably sufficient for the maintenance of the metabolic phenotype in adult offspring.

Thus, findings from our study show that rats exposed to overnutrition during early stages of life via maternal diet are predisposed to obesity throughout their life. One of the mechanisms behind this programming effect is epigenetic modifications in *Pomc* regulatory regions. These modifications would prevent a response to leptin-mediated stimulation of *Pomc* expression and thereby affect energy homeostasis. Moreover, these offspring are programmed to have an exacerbated response to postweaning HF diet challenge resulting in metabolic disorders. Although additional research is needed to understand whether the programmed obesity phenotype could be reprogrammed by postweaning interventions, our results demonstrate that the perinatal developmental period serves as a critical time window during which a mother’s exposure to excess calories can influence lifelong health and behavior of the offspring. Given that the rate of obesity in women of child-bearing age is increasing alarmingly in the western world [51–53], and the offspring of these obese mothers are predisposed to being overweight or obese [54–56], these results indicate that epigenetic malprogramming during critical developmental periods could be one of the factors affecting the increasing obesity worldwide.

## Electronic supplementary material

Supplementary figure legends and supplementary table

Maternal obesity

Mean relative gene expression levels of orexigeneic neuropeptides in offspring at 3 weeks of age and at adulthood

Mean relative gene expression levels of DNA methylation related genes in offspring

DNA methylation changes at hypothalamic Pomc distal promoter regions in 3 and 20 week-old offspring
