# Supplementary material for: Maternal overnutrition programs epigenetic changes in the regulatory regions of hypothalamic Pomc in the offspring of rats
Source: Int J Obes (Lond). 2018 May 17;42(8):1431–44. doi: 10.1038/s41366-018-0094-1 (PMC6113193; doi:10.1038/s41366-018-0094-1)
Supplement: Supplementary file 1 — Supplementary figure legends and supplementary table [file 41366_2018_94_MOESM1_ESM.docx]

**Figure S1: Maternal obesity.** (A) Maternal body weight was measured 6 weeks prior to conception, throughout gestation and lactation. (B) Calorific intake from fat for dams on LF or HF diet during pre-pregnancy, pregnancy and lactation. (C) Average daily calorific intake. (D) Mesenteric and subcutaneous fat mass and (E) plasma leptin levels in LF or HF diet fed dams at the end of lactation. Data were analysed by Student’s t-test, n=8 and are shown as mean + SEM. *P<0.05, ****P<0.0001.

**Figure S2: Mean relative gene expression levels of orexigeneic neuropeptides in offspring at 3 weeks of age and at adulthood.** (A) mRNA expression levels of *galanin*, *enkephalin* and *dynorphin* in ARC analysed by qRT-PCR in the offspring of LF or HF fed mothers at 3 weeks (n=8) and (B) that of *galanin* and *enkephalin* in adult offspring fed postnatally LF or HF diet (n=6). Data are shown as mean + SEM.

**Figure S3: Mean relative gene expression levels of DNA methylation related genes in offspring.** (A) mRNA expression levels of *Dnmt1*, *Dnmt3a*, *Mecp2* and *Mbd2* in ARC analysed by qRT-PCR in the offspring of LF or HF fed mothers at weaning (n=8) and (C) in adult offspring fed postnatally LF or HF diet (n=6). (B) mRNA expression levels of SP1 in ARC of offspring of LF or HF fed mothers at weaning (n=8) and (D) in adult offspring fed postnatally LF or HF diet (n=6). Data are shown as mean + SEM. *Dnmt1*, DNA methyltransferase 1; *Dnmt3a*, DNA methyltransferase 3a; *Mecp2*, Methyl CpG binding protein 2; *Mbd2*, methyl-CpG binding domain protein 2 and SP1, Specificity protein 1.

**Figure S4: DNA methylation changes at hypothalamic *Pomc* distal promoter regions in 3 and 20 week-old offspring.** (A) Methylation analyses of hypothalamic *Pomc* promoter (-250 to -150 bp) in the offspring of LF or HF fed mothers at weaning (n=8) and (B) in adult offspring fed postnatally LF or HF diet. -224 site (two-way ANOVA: ^‡^Post-weaning diet effect, P=0.0335; interaction, P= 0.0076, Post-hoc Tukey, n=6), -202 site (two-way ANOVA: Maternal diet effect, ^†^P=0.0428, n=6). Data are shown as mean + SEM. **P<0.01.

Supplementary Table 1

| **Gene** | Forward primer | Reverse primer |
| --- | --- | --- |
| Pomc | GCTACGGCGGCTTCATGA | CCTCACTGGCCCTTCTTGTG |
| AgRP | AGCAGACCGAGCAGAAGATG | GACTCGTGCAGCCTTACACA |
| Npy | TCCTAGTTTCCCCCCACATCT | AAGGGAAATGGGTCGGAATC |
| Ob-Rb | CCAGTACCCAGAGCCAAAGT | GGGCTTCACAACAAGCATGG |
| MC4R | ACGGGTCAGAAACCATCGTC | GCGAGCAAGGAGCTACAGAT |
| NPY1R | CAACCCAAGAGGGTGGAGAC | ACGAAGGGCAGAGAAGAAGC |
| Galanin | TTC CCA CCA CTG CTC AAG ATG | TGG CTG ACA GGG TTG CAA |
| Enkephalin | GGA CTG CGC TAA ATG CAG CTA | GTG TGC ATG CCA GGA AGT TG |
| Dynorphin | TCAACCCCCTGATTTGCTCC | GGCAGTCAGGGTGAGAAAAGA |
| Dnmt1 | CAATCCCAAAGCTCAACCCG | CATTGGTCAGCATCTGGGGT |
| Dnmt 3a | CCCACAGCGCCCTCGAA | CCTTCCTTTCGATCATCCTCCC |
| Mecp2 | GGGCTCAGGGAGGAAAAGTC | AGTGGTTCATGTTTGCCCTCT |
| Mbd2 | CCCCTCAATCAGAACAAGGGTAA | CGGGTGGTTTGTGACTTTGG |
| SP1 | ACCATGAGCGACCAAGATCA | TCGAGTCTGAGAAAAGGCGG |
| HPRT | TGGTCAAGCAGTACAGCCCC | TACTGGCCACATCAACAGGA |
