# Supplementary figures and images for: Maternal overnutrition programs epigenetic changes in the regulatory regions of hypothalamic Pomc in the offspring of rats
Source: Int J Obes (Lond). 2018 May 17;42(8):1431–44. doi: 10.1038/s41366-018-0094-1 (PMC6113193; doi:10.1038/s41366-018-0094-1)

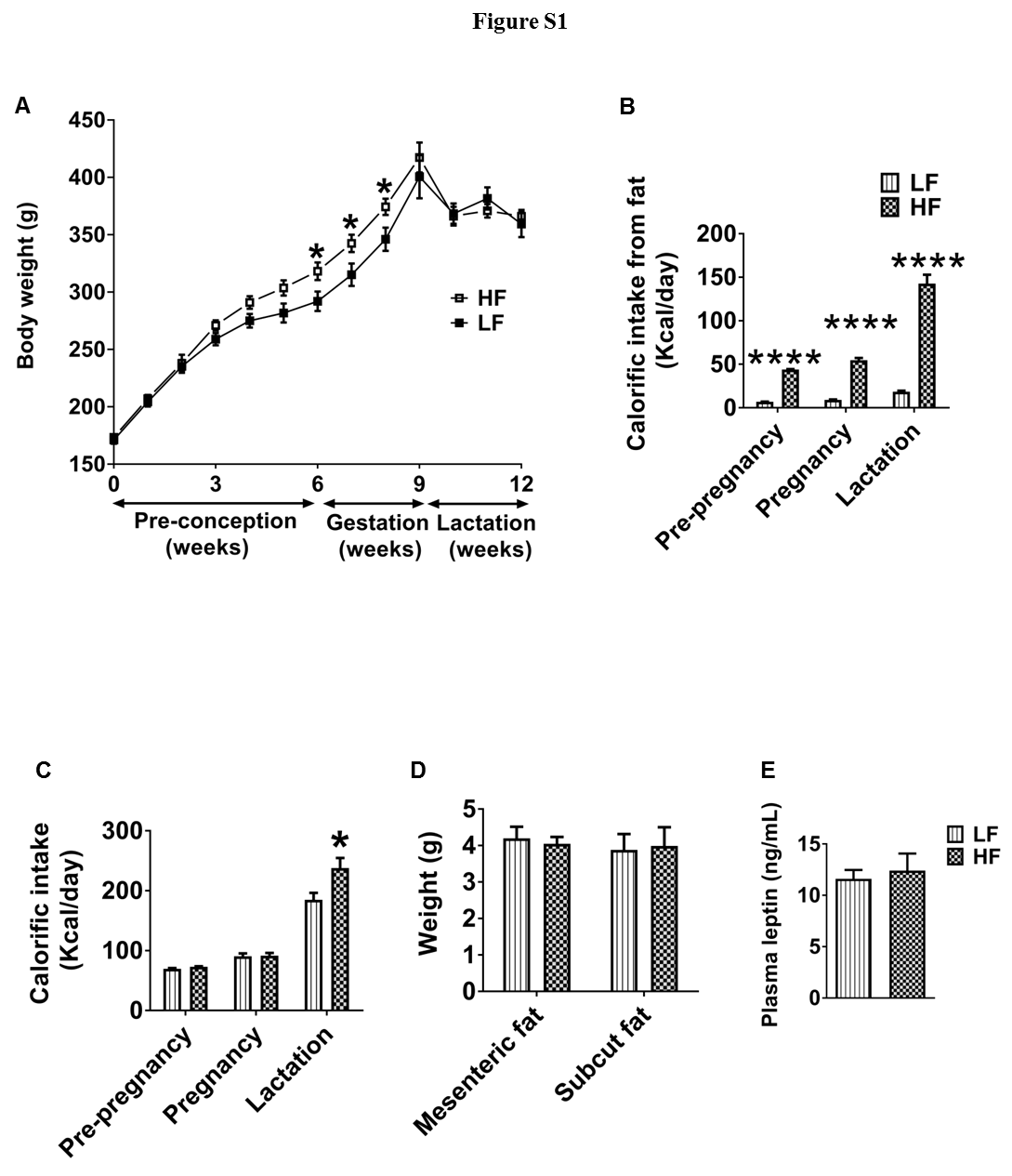

Supplement: Supplementary file 2 — Maternal obesity [file 41366_2018_94_MOESM2_ESM.tif]

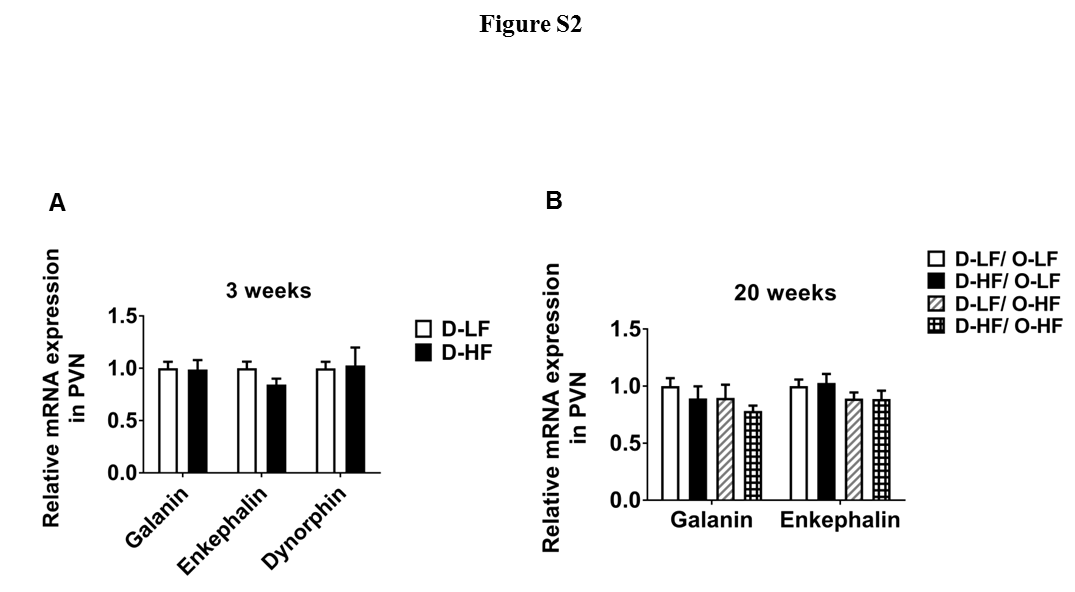

Supplement: Supplementary file 3 — Mean relative gene expression levels of orexigeneic neuropeptides in offspring at 3 weeks of age and at adulthood [file 41366_2018_94_MOESM3_ESM.tif]

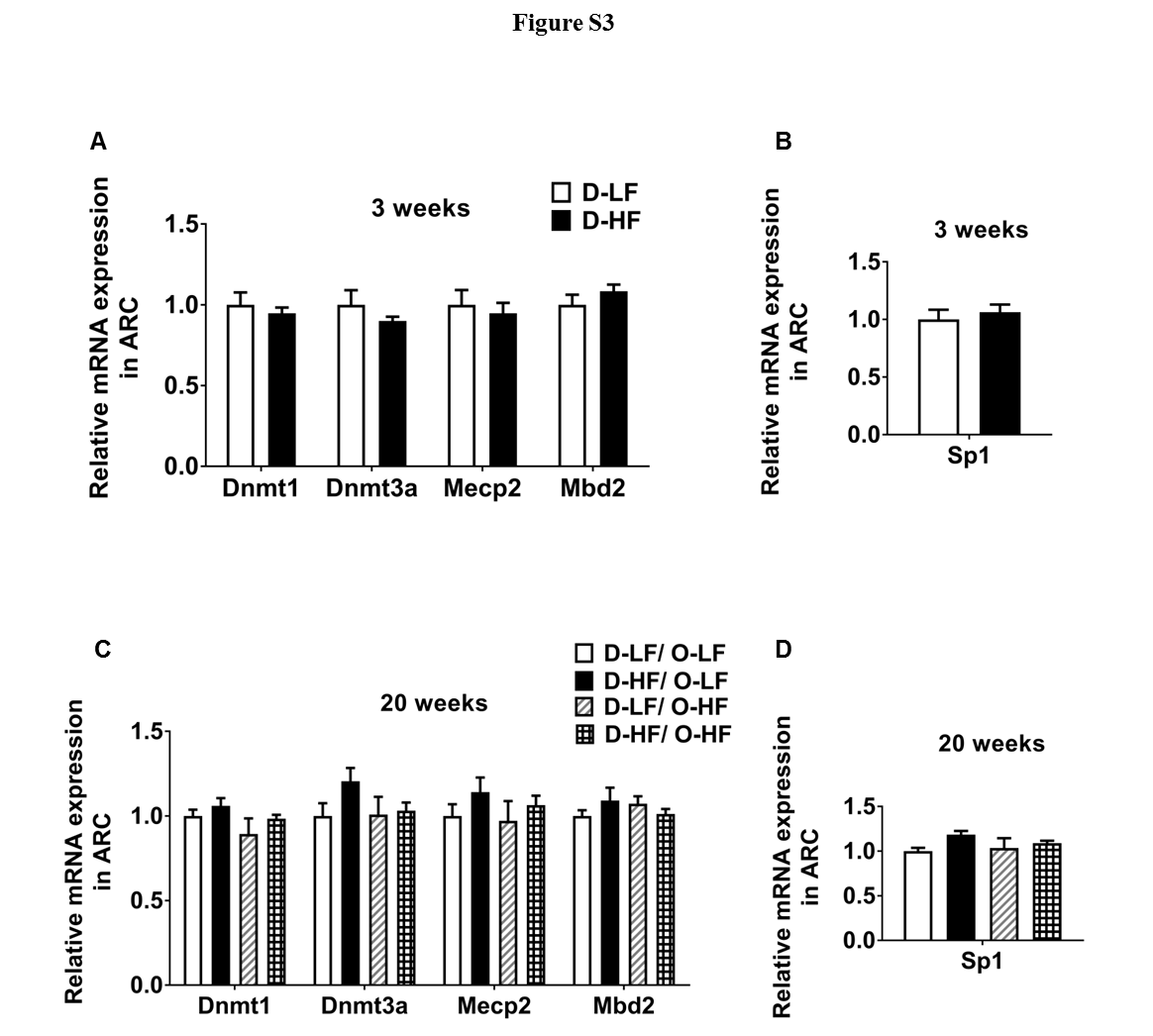

Supplement: Supplementary file 4 — Mean relative gene expression levels of DNA methylation related genes in offspring [file 41366_2018_94_MOESM4_ESM.tif]

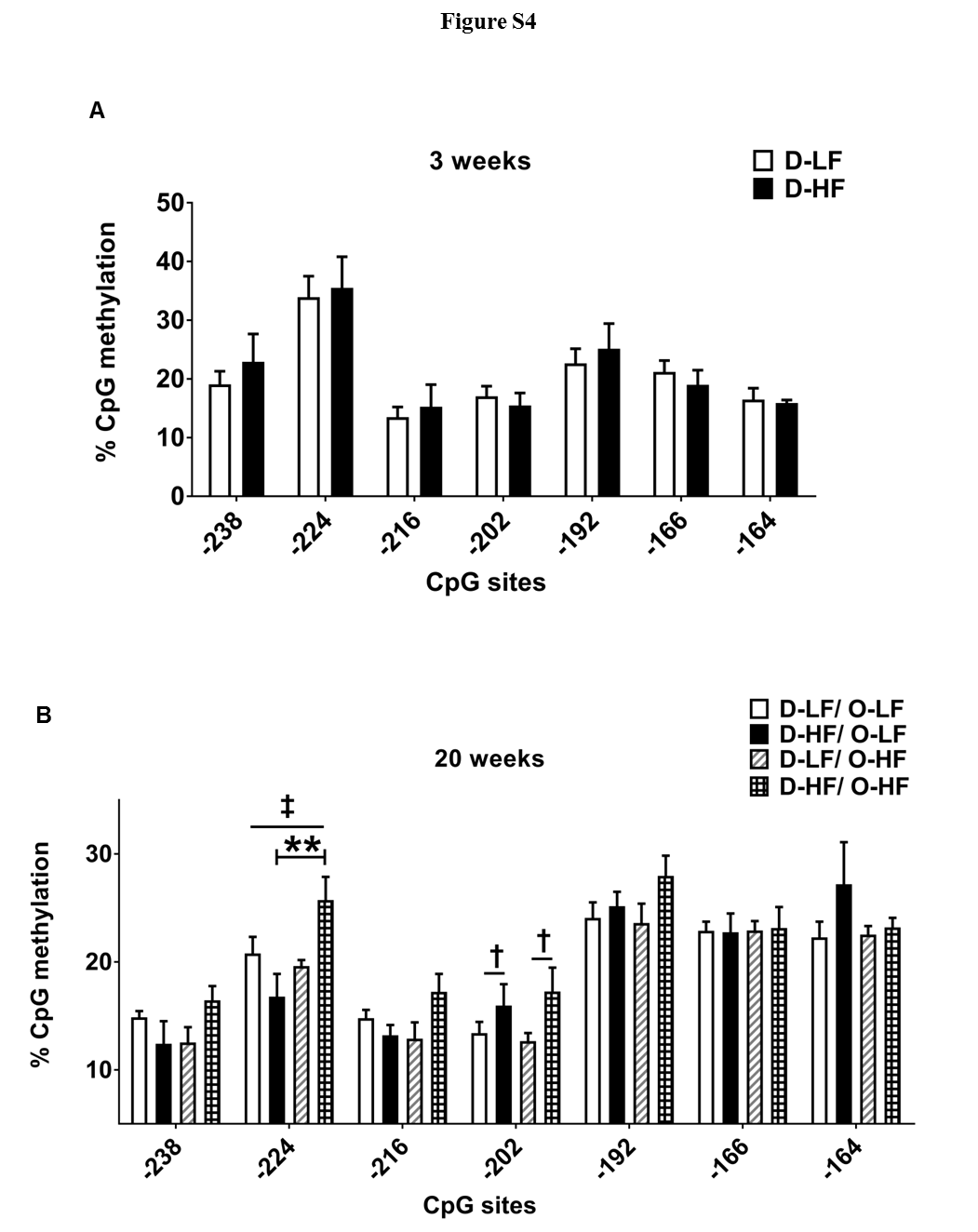

Supplement: Supplementary file 5 — DNA methylation changes at hypothalamic Pomc distal promoter regions in 3 and 20 week-old offspring [file 41366_2018_94_MOESM5_ESM.tif]
